# Trends in Use and Perceptions About Triplet Chemotherapy Plus Bevacizumab for Metastatic Colorectal Cancer

**DOI:** 10.1001/jamanetworkopen.2021.24766

**Published:** 2021-09-10

**Authors:** Sietske C. van Nassau, Marinde J. Bond, Ilva Scheerman, Jesper van Breeschoten, Rob Kessels, Liselot B. Valkenburg-van Iersel, Henk M. Verheul, Tineke E. Buffart, Leonie J. Mekenkamp, Valery E. Lemmens, Miriam Koopman, Guus M. Bol

**Affiliations:** 1 Department of Medical Oncology, Division Cancer Center and Imaging, University Medical Center Utrecht, Utrecht, the Netherlands; 2 Department of Epidemiology, Julius Center for Health Sciences and Primary Care, University Medical Center Utrecht, Utrecht, the Netherlands; 3 Department of Medical Oncology, Amsterdam University Medical Center, Vrije Universiteit Medical Center, Cancer Center Amsterdam, Amsterdam, the Netherlands; 4 Dutch Oncology Research Platform, Antoni van Leeuwenhoek Hospital, Amsterdam, the Netherlands; 5 Division of Medical Oncology, Department of Internal Medicine, GROW–School for Oncology and Developmental Biology, Maastricht University Medical Center, Maastricht, the Netherlands; 6 Department of Medical Oncology, Radboud University Medical Center, Nijmegen, the Netherlands; 7 Department of Gastrointestinal Oncology, Netherlands Cancer Institute, Amsterdam, the Netherlands; 8 Department of Medical Oncology, Medisch Spectrum Twente, Enschede, the Netherlands; 9 Board of Directors, Netherlands Comprehensive Cancer Organisation, Utrecht, the Netherlands; 10 Department of Public Health, Erasmus University Medical Centre, Rotterdam, the Netherlands

## Abstract

**Importance:**

Triplet chemotherapy with fluorouracil, folinic acid, oxaliplatin, and irinotecan plus bevacizumab (FOLFOXIRI-B) is an effective first-line treatment option for patients with metastatic colorectal cancer (mCRC). However, the degree of implementation of FOLFOXIRI-B in daily practice is unknown.

**Objectives:**

To evaluate the current adoption rate of FOLFOXIRI-B in patients with mCRC and investigate the perspectives of medical oncologists toward this treatment option.

**Design, Setting, and Participants:**

This 1-week, multicenter, cross-sectional study in the Netherlands used a flash mob design, which facilitates ultrafast data generation (flash) through the engagement of numerous researchers (mob). During the study week (March 1-5, 2021), patient data were retrieved from electronic health records of 47 hospitals on patients with mCRC who were referred to a medical oncologist between November 1, 2020, and January 31, 2021. Interviews were simultaneously conducted with 101 medical oncologists from 52 hospitals who regularly treat patients with mCRC.

**Exposure:**

First-line systemic treatment as determined by the treating physician.

**Main Outcomes and Measures:**

The FOLFOXIRI-B prescription rate was the main outcome. Current practice was compared with prescription rates in 2015 to 2018. Eligibility for treatment with FOLFOXIRI-B was estimated. An exploratory outcome was medical oncologists’ reported perspectives on FOLFOXIRI-B.

**Results:**

A total of 5948 patients in the Netherlands (median age [interquartile range], 66 [57-73] years; 3503 [59%] male; and 3712 [62%] with left-sided or rectal tumor) were treated with first-line systemic therapy for synchronous mCRC. A total of 282 patients with mCRC underwent systemic therapy during the study period (2021). Of these 282 patients, 199 (71%) were treated with intensive first-line therapy other than FOLFOXIRI-B, of whom 184 (65%) were treated with oxaliplatin doublets with or without bevacizumab; 14 (5%) with irinotecan doublets with or without bevacizumab, panitumumab, or cetuximab; and 1 (0.4%) with irinotecan with bevacizumab. Fifty-four patients (19%) were treated with fluoropyrimidine monotherapy with or without bevacizumab, 1 patient (0.4%) with panitumumab monotherapy, and 3 (1%) with immune checkpoint inhibitors. In total, 25 patients (9%; 95% CI, 6%-12%) were treated with first-line FOLFOXIRI-B compared with 142 (2%; 95% CI, 2%-3%) in 2015 to 2018. During the study period, 21 of 157 eligible patients (13.4%) in the Netherlands were treated with FOLFOXIRI-B. A total of 87 medical oncologists (86%) reported discussing FOLFOXIRI-B as a treatment option with eligible patients. A total of 47 of 85 (55%) generally communicated a preference for a chemotherapy doublet to patients. These oncologists reported a significantly lower awareness of guidelines and trial results. Toxic effects were the most reported reason to prefer an alternative regimen.

**Conclusions and Relevance:**

The findings of this study suggest that FOLFOXIRI-B prescription rates have marginally increased in the last 5 years. Considering that most medical oncologists discuss this treatment option, the prescription rate found in this study was below expectations. Awareness of guidelines and trial data seems to contribute to the discussion of available treatment options by medical oncologists, and the findings of this study suggest a need for repeated and continuing medical education.

## Key Points

**Question:**

What is the current rate of adoption of first-line systemic treatment with fluorouracil, folinic acid, oxaliplatin, and irinotecan plus bevacizumab (FOLFOXIRI-B) in daily practice?

**Findings:**

In this cross-sectional study of 282 patients in the Netherlands, FOLFOXIRI-B prescription rates marginally increased in the past 5 years. During the study period, 1 in 7 estimated, eligible patients was treated with FOLFOXIRI-B.

**Meaning:**

The results of this study suggest that, despite evidence of the effectiveness of FOLFOXIRI-B therapy, use of this therapy for metastatic colorectal cancer remains low, possibly because of oncologists’ reported preference for doublet therapy.

## Teaser

This cross-sectional study compares current rates of prescription of fluorouracil, folinic acid, oxaliplatin, and irinotecan plus bevacizumab (FOLFOXIRI-B) with rates from 2015 to 2018 and assesses Dutch medical oncologists’ perspectives on treatment with FOLFOXIRI-B.

## Introduction

In the past 2 decades, the choice of first-line treatment of patients with metastatic colorectal cancer (mCRC) has become more complex because of the increased availability of treatment options.^1^ A novel first-line systemic treatment option for fit patients, who are candidates for intensive chemotherapy, is fluorouracil, folinic acid, oxaliplatin, and irinotecan plus bevacizumab (FOLFOXIRI-B). The efficacy and safety of FOLFOXIRI-B have been assessed by several studies, including 2 phase 3 randomized clinical trials.^2,3,4^ In the TRIBE (Combination Chemotherapy and Bevacizumab as First-Line Therapy in Treating Patients With Metastatic Colorectal Cancer) trial, FOLFOXIRI-B was compared with fluorouracil, folinic acid, irinotecan, and bevacizumab (FOLFIRI-B), showing an improvement in progression-free survival of 2.6 months and, as a secondary end point, improved overall survival of 4 months.^2,3^ Since publication of the TRIBE trial in 2014, the triplet regimen plus bevacizumab has been incorporated into national and international guidelines.^1,5,6,7,8^

Additional evidence for FOLFOXIRI-B was recently provided by the TRIBE2 (Upfront FOLFOXIRI Plus Bevacizumab and Reintroduction After Progression Versus mFOLFOX6 Plus Bevacizumab Followed by FOLFIRI Plus Bevacizumab in the Treatment of Patients With Metastatic Colorectal Cancer) trial in 2020,^4^ which demonstrated that upfront FOLFOXIRI-B, followed by reintroduction after first disease progression, was superior to sequential administration of fluorouracil, oxaliplatin, and folinic acid plus bevacizumab (FOLFOX-B) and FOLFIRI-B (statistically significant benefit in progression-free survival 2 of 2.8 months and, as a secondary end point, in overall survival of 4.9 months). The efficacy of this regimen was confirmed in a meta-analysis^9^ that included all available data from randomized clinical trials. This regimen was subsequently approved in 2020 by Dutch health care authorities.^10^ No subgroup has been identified for which no benefit is expected,^9^ although efficacy may be compromised in patients previously treated with oxaliplatin-based adjuvant chemotherapy. Because FOLFOXIRI-B has not been prospectively compared to doublet chemotherapy plus an antibody to the epidermal growth factor receptor in patients with left-sided primary and *RAS *(OMIM 164790, 190070, and 19002090070)*/BRAF^V600^* (OMIM 164757) wild-type tumors, its benefit in this subgroup is uncertain.

Compared with a doublet chemotherapy backbone, FOLFOXIRI-B leads to a higher incidence of grades 3 to 4 diarrhea, (febrile) neutropenia, nausea, and mucositis.^9^ Furthermore, FOLFOXIRI-B treatment involves the need for a central venous catheter and biweekly schedule. The latter is a relevant difference to the frequently used, 3-weekly capecitabine-based regimen. The published data justify FOLFOXIRI-B as a first-line treatment for eligible patients. However, the benefit-burden tradeoff presumably differs per individual.

In this study, we assess the adoption of FOLFOXIRI-B treatment for patients with mCRC in clinical practice in 3 parts. First, we assess the prescription of FOLFOXIRI-B from January 1, 2015, to December 31, 2018. Second, we explore the current prescription of FOLFOXIRI-B (November 1, 2020, to January 31, 2021). To provide a true representation of current practice, we used a cross-sectional flash mob study design. A flash mob is defined as “a group of people summoned to a designated location at a specified time to perform an indicated action before dispersing.”^11^ Using this concept as a research design allows us to maximize the number of patients, physicians, and hospitals represented within a short time frame.^12,13,14,15,16,17^ Third, we investigate Dutch medical oncologists’ perspectives on treatment with FOLFOXIRI-B.

## Methods

### Study Design

The objective of this cross-sectional study was to assess the adoption of FOLFOXIRI-B as a first-line systemic regimen for mCRC patients in daily practice. For this purpose, we made use of 3 data sources: the Netherlands Cancer Registry (NCR), patient data collected over the course of 1 week from 47 Dutch hospitals, and interviews with medical oncologists (eFigure 1 in Supplement 1). All data were deidentified. The study protocol was approved by the local institutional review boards at 41 participating sites. In addition, the study was approved by the scientific council of the Netherlands Comprehensive Cancer Organization. The need to obtain patient informed consent was waived according to Dutch laws and regulations. This study followed the Strengthening the Reporting of Observational Studies in Epidemiology (STROBE) reporting guideline.^18^

### NCR Patient Data, 2015-2018

We first assessed prescription rates of FOLFOXIRI-B and other first-line systemic therapy regimens in a retrieved mCRC cohort from the NCR. The NCR is a population-based registry that covers the total Dutch population. All NCR data are collected from medical records by trained data managers. Our cohort of patients with mCRC consisted of patients who were diagnosed with stage IV (synchronous) mCRC between January 1, 2015, and December 31, 2018, and subsequently started first-line systemic therapy. Data on metachronous metastases are not included in the NCR.

### Flash Mob–Collected Patient Data, March 2021

To assess the current adoption of FOLFOXIRI-B, we conducted a nationwide, 1-week, multicenter, cross-sectional observational study, also known as a flash mob design. All Dutch hospitals (n = 72) were invited to participate in this flash mob study through members of the Dutch Society of Medical Oncology (NVMO) and the Dutch Colorectal Cancer Group. In total, 47 of the 72 medical centers participated. Six of the 47 medical centers could not comply with timely regulatory requests. In these centers, data were collected by trained data managers from the NCR.

All patients with a new diagnosis of mCRC who were referred to a medical oncologist in participating centers between November 1, 2020, and January 31, 2021, were included if first-line systemic therapy was administered before March 6, 2021. During the study week (March 1 to March 5, 2021), clinicopathological data, including age, sex, Eastern Cooperative Oncology Group (ECOG) performance status (PS), tumor sidedness, molecular characteristics, time to metastasis, location of metastasis, primary tumor resection, previous exposure to oxaliplatin-based adjuvant chemotherapy, first-line clinical trial participation, and first-line systemic therapy, were collected from electronic health records by internal medicine residents, oncology nurses, and medical interns under the supervision of a medical oncologist (G.M.B., L.B.V., H.M.V., T.E.B. and L.J.M.), all of whom were local to the participating center. Patient records were anonymized and variables were entered into electronic case report forms (Castor). All data collectors were trained to collect the data according to prespecified criteria.

When time from initial diagnosis to the occurrence of metastasis was less than 6 months, patients were classified as having synchronous metastatic disease and, if 6 months or more, as having metachronous metastatic disease.^19,20^ We estimated which patients would have been eligible for treatment with FOLFOXIRI-B. We based our definition for estimated eligibility on the TRIBE trials’ eligibility criteria: patients 75 years or younger with an ECOG PS of 0 to 2 who were treated with oxaliplatin doublets (ie, oxaliplatin, fluorouracil, and folinic acid or capecitabine and oxaliplatin) or FOLFOXIRI with or without bevacizumab. We chose to limit the definition to oxaliplatin-based systemic treatment to exclude patients who would not have been eligible because of oxaliplatin contraindications, such as peripheral neuropathy. Patients in clinical trials with systemic treatment were excluded. We pooled patients treated with FOLFOXIRI with and without bevacizumab. Therefore, further mention of FOLFOXIRI-B in this article also includes FOLFOXIRI (eMethods in Supplement 1).

### Interviews With Medical Oncologists

All medical oncologists who treat patients with mCRC in the Netherlands were invited by email or a direct colleague for an interview during the study week. Medical oncologists were initially not informed about the exact research question of this study to prevent information bias. They were simply informed that the interview would concern their personal approach to first-line systemic treatment choices for patients with mCRC. Interviews were conducted by medical oncologists, PhD degree candidates, and postdoctoral researchers with a focus on CRC-related research. All interviewers were trained to perform the interviews according to the specific interview script (eTable 2 and eFigure 2 in Supplement 1). The interview script was authored by 6 medical oncologists who specialized in CRC (M.K., T.E.B., L.B.V., H.M.V., G.M.B., and L.J.M.) and reviewed by an epidemiologist. To minimize interviewer bias, the interview script existed predominantly of closed questions. For the semi–open-ended questions, interviewers were strictly instructed to classify the answers to predetermined answer categories. If none of the predetermined answers were appropriate according to the interviewer, the answer was centrally reviewed (S.C.N. and G.M.B.) and classified if possible. Interview records were anonymized and immediately entered into the electronic data capture tool (Castor). For every interview, a FOLFOXIRI-B Awareness Score (FAS) was calculated. FAS is a combined score for awareness of the guidelines, clinical trials, and NVMO-committee recommendation (eMethods in Supplement 1). FAS represents the medical oncologist’s awareness of FOLFOXIRI-B in multiple scientific materials. A lower FAS score means less awareness of the FOLFOXIRI-B literature.

### Statistical Analysis

Data were exported from the electronic case report form to SPSS, version 27.0 (SPSS Inc). Descriptive statistics were used to present baseline characteristics. A 2-tailed, unpaired *t* test was used to compare continuous variables (age and FAS) between groups. A χ^2^ test was used for group comparison of categorical variables. When groups were too small, we used the Fisher exact test. A 2-sided *P* < .05 was considered statistically significant.

## Results

### NCR Clinical Data, 2015-2018

From 2015 to 2018, a total of 5948 patients (median age [interquartile range], 66 [57-73] years; 3503 [59%] male; and 3712 [62%] with left-sided or rectal tumor) were treated with first-line systemic therapy for synchronous mCRC in the Netherlands. Metastases were confined to the liver in 2277 patients (38%) of patients, and a *RAS* variant (OMIM 164790, 190070, and 190020) was present in 1514 of 2948 patients (51%) and a *BRAF^V600^* variant in 392 of 2560 patients (15%). Baseline characteristics are given in eTable 1 in Supplement 1. Of the total of 5948 patients, 1281 (22%) were treated with capecitabine or fluorouracil monotherapy with or without bevacizumab, 4286 (72%) with oxaliplatin-based doublets with or without bevacizumab, 49 (0.8%) with irinotecan monotherapy with or without bevacizumab, and 142 (2%) with FOLFOXIRI or FOLFOXIRI-B, of whom 71 (50%) were enrolled in a clinical trial that involved the use of FOLFOXIRI-B. An additional 190 patients received other treatment regimens, including anti-eGFR therapy and immunotherapy (eTable 1 in Supplement 1). According to our eligibility estimate, 3696 (62%) were estimated to be eligible for treatment with FOLFOXIRI-B, of whom 71 (1.9%) were treated with FOLFOXIRI-B.

### Flash Mob Clinical Data, 2020-2021

From March 1 to March 5, 2021, clinical data were collected from 402 patients with mCRC in 47 of a total of 72 Dutch hospitals, of whom 120 patients did not meet study eligibility criteria and were excluded from analysis (eFigure 1 in Supplement 1). Baseline characteristics are given in eTable 1 in Supplement 1. Of the 282 included patients (median [interquartile range] age, 66 [57-73] years, 164 [58%] male; and 235 [83%] with an ECOG PS of 0-1), 199 patients (71%) were treated with intensive first-line therapy other than FOLFOXIRI-B, of whom 184 (65%) were treated with oxaliplatin doublets with or without bevacizumab; 14 (5.0%) with irinotecan doublets with or without bevacizumab, panitumumab, or cetuximab; and 1 (0.4%) with irinotecan with bevacizumab. Fifty-four patients (19%) were treated with fluoropyrimidine monotherapy with or without bevacizumab, 1 (0.4%) with panitumumab monotherapy, and 3 (1%) with immune checkpoint inhibitors. In total, 25 (9%) were treated with FOLFOXIRI-B. Four of these patients received FOLFOXIRI-B within the scope of a clinical trial (Table 1).

**Table 1.** Characteristics of 282 Patients Treated With First-Line Systemic Therapy in 2020-2021^a^

Characteristic	FOLFOXIRI-B (n = 25)	Other intensive treatment (n = 199)^b^	Monotherapy (n = 58)^c^
Age, y			
Median (IQR)	59 (52-64)	65 (55-72)	76 (68-81)
≤75	25 (100)	168 (84)	29 (50)
>75	0	31 (16)	29 (50)
Sex			
Male	18 (72)	113 (57)	33 (57)
Female	7 (28)	86 (43)	25 (43)
ECOG performance status			
0-1	25 (100)	167 (84)	43 (74)
2	0	17 (8)	9 (16)
3	0	4 (2)	4 (7)
Missing	0	11 (6)	2 (3)
Primary tumor site			
Right sided	9 (36)	69 (35)	20 (35)
Left sided or rectum	16 (64)	125 (63)	36 (62)
Unknown or not specified	0	5 (2)	2 (3)
Resected primary tumor			
Yes	9 (36)	75 (38)	29 (50)
No	16 (64)	124 (62)	29 (50)
Time to metastases			
Synchronous	20 (80)	152 (76)	35 (60)
Metachronous	5 (20)	47 (24)	23 (40)
Liver only			
Yes	13 (52)	52 (26)	5 (9)
No	12 (48)	147 (74)	53 (91)
*BRAF^V600^* status			
Wild type	17 (68)	126 (63)	25 (43)
Variant	4 (16)	19 (10)	3 (5)
Missing	4 (16)	54 (27)	30 (52)
*RAS* status			
Wild type	11 (44)	70 (35)	10 (17)
Variant	10 (40)	76 (38)	18 (31)
Missing	4 (16)	53 (27)	30 (52)
Microsatellite status			
MSS or proficient MMR	22 (88)	147 (74)	25 (43)
MSI or deficient MMR	0	5 (2)	6 (10)
Missing	3 (12)	47 (24)	27 (47)
Systemic first-line therapy			
Oxaliplatin based	25 (100)	184 (92)	0
Irinotecan based	25 (100)	15 (8)	0
Systemic first-line included bevacizumab	22 (88)	160 (80)	35 (60)
Prior adjuvant chemotherapy	4 (16)	13 (6)	6 (10)
Participation in first-line clinical trial^d^	4 (16)	22 (11)	3 (5)

Abbreviations: ECOG, Eastern Cooperative Oncology Group; FOLFOXIRI-B, fluorouracil, folinic acid, oxaliplatin, and irinotecan plus bevacizumab; IQR, interquartile range; MMR, mismatch repair; MSI, microsatellite instable; MSS, microsatellite stable.

^a^ Data are presented as number (percentage) of patients unless otherwise indicated.

^b^ Intensive systemic therapy was defined as irinotecan monotherapy or capecitabine and oxaliplatin; fluorouracil, oxaliplatin, and folinic acid; or fluorouracil, folinic acid, and irinotecan with or without biological.

^c^ Capecitabine and fluorouracil with or without biological and immune checkpoint inhibition.

^d^ Trials included Capecitabine, Irinotecan, Oxaliplatin (CAIRO) 4, 5, and 6 trials; Chemotherapy and Maximal Tumor Debulking of Multi-organ Colorectal Cancer Metastases (ORCHESTRA) trial; Intensive Blood Pressure Reduction in Acute Cerebral Hemorrhage Trial (INTERACT); Pressurized Intraperitoneal Aerosol Chemotherapy (PIPAC) II trial; Study of Nivolumab, Nivolumab Plus Ipilimumab, or Investigator's Choice Chemotherapy for the Treatment of Participants With Deficient Mismatch Repair/Microsatellite Instability High Metastatic Colorectal Cancer (CheckMate 8HW); Hepatic Arterial Infusion Pump Chemotherapy Combined With Systemic Chemotherapy (PUMP-IT) trial; and Drug Rediscovery Protocol (DRUP) trial.

Since 2015 to 2018 (data source 1), FOLFOXIRI-B prescription increased from 2% (95% CI, 2%-3%) to 9% (95% CI, 6%-12%) in 2020 to 2021 (data source 2). We estimated that, currently (data source 2), 157 of 282 patients (56%) were eligible for treatment with FOLFOXIRI-B (Table 2). Twenty-one of these patients (13%) received FOLFOXIRI-B treatment. Patients who were treated with first-line FOLFOXIRI-B were younger (median age, 58 vs 62 years; *P* = .006) and more frequently treated with adjuvant oxaliplatin-based chemotherapy (19% vs 4%; *P* = .03) compared with estimated eligible patients not treated with FOLFOXIRI-B. Notable absolute differences were seen in ECOG PS, liver-only metastasis, and *BRAF* status; however, these findings were not significantly different.

**Table 2.** Differences Between Patients Treated With FOLFOXIRI-B Compared With Estimated Eligible Patients^a^

Characteristic	FOLFOXIRI-B (n = 21)^b^	Estimated eligible patients not treated with FOLFOXIRI-B (n = 136)^c^	*P* value^d^
Age, median (IQR), y	58 (50-64)	62 (56-70)	.006^e^
Sex			
Male	16 (76)	79 (58)	.14
Female	5 (24)	57 (42)	
ECOG performance status			
0-1	21 (100)	113 (83)	.13^f^
2	0	15 (11)	
Missing	0	8 (6)	
Primary tumor site			
Right sided	7 (33)	44 (32)	.73
Left sided or rectum	14 (67)	88 (65)	
Not specified	0	4 (3)	
Resected primary tumor			
Yes	12 (57)	54 (40)	.78
No	9 (43)	82 (60)	
Time to metastases			
Synchronous	16 (76)	106 (78)	.86
Metachronous	5 (24)	30 (22)	
Liver only			
Yes	9 (43)	41 (30)	.31
No	12 (57)	95 (70)	
*BRAF* status			
Wild type	13 (62)	84 (62)	.30^f^
Variant	4 (19)	14 (10)	
Missing	4 (19)	38 (28)	
*RAS* status			
Wild type	10 (48)	40 (29)	.17
Variant	7 (33)	58 (43)	
Missing	4 (19)	38 (28)	
Microsatellite status			
MSS or proficient MMR	18 (86)	103 (76)	>.99^f^
MSI or deficient MMR	0	3 (2.2)	
Missing	3 (14)	30 (22)	
Prior adjuvant chemotherapy	4 (19)	6 (4)	.03^f^
Systemic first-line included bevacizumab	18 (86)	110 (81)	.77^f^

Abbreviations: ECOG, Eastern Cooperative Oncology Group; eGFR, estimated glomerular filtration rate; FOLFOXIRI-B, fluorouracil, folinic acid, oxaliplatin, and irinotecan plus bevacizumab; IQR, interquartile range; MMR, mismatch repair; MSI, microsatellite instable; MSS, microsatellite stable.

^a^ Data are presented as number (percentage) of patients unless otherwise indicated.

^b^ The number of patients treated with FOLFOXIRI-B is different from the number reported in Table 1. This total is due to the exclusion of 4 patients who were treated in a clinical trial to prevent bias in the comparison (see estimated eligibility definition below).

^c^ Estimated eligibility was defined as: patients ≤75 years of age with an ECOG performance status of 0-2, who were treated with FOLFOXIRI (fluorouracil, folinic acid, oxaliplatin, and irinotecan) or oxaliplatin-doublets (ie, FOLFOX [fluorouracil, folinic acid, and oxaliplatin] or CAPOX [capecitabine and oxaliplatin]) with or without bevacizumab outside of a clinical trial. There were no patients treated with anti-eGFR antibodies and an oxaliplatin doublet in data source 2.

^d^ χ^2^ Tests were performed unless otherwise indicated.

^e^ *t* Test.

^f^ Fisher exact test.

### Interviews With Medical Oncologists

In total, 101 medical oncologists (median [interquartile range] age, 43 [38-52] years; 49 [48%] male; and 17 [17%] working at an academic hospital or specialized cancer center) working at 52 different hospitals and involved in the treatment of patients with mCRC agreed to be interviewed in the study week. Baseline characteristics of medical oncologists are presented in Table 3.

**Table 3.** Characteristics of the Interviewed Medical Oncologists^a^

Characteristic	All medical oncologists (N = 101)	Medical oncologists who discuss FOLFOXIRI-B with patients (n = 87)	Medical oncologists who do not discuss FOLFOXIRI-B with patients (n = 14)	*P* value^b^
Age, y				
Median (IQR)	43 (38-52)	42 (38-50)	53 (38-56)	.10^c^
≤40	38 (38)	34 (39)	4 (29)	
>40	63 (62)	53 (61)	10 (71)	
Sex				
Male	49 (48)	37 (43)	12 (86)	.003
Female	52 (52)	50 (57)	2 (14)	
Practice setting				
Academic hospital^d^	17 (17)	17 (20)	0	.12^e^
Teaching hospital	57 (56)	48 (55)	9 (64)	
Community hospital	27 (27)	22 (25)	5 (36)	
Duration of practice, y				
≤3	24 (24)	23 (26)	1 (7)	.18
>3	77 (76)	64 (74)	13 (93)	
No. of new patients with mCRC treated by an oncologist per year				
High volume (≥25)	52 (52)	42 (48)	10 (71)	.11
Low volume (12-24)	49 (48)	45 (52)	4 (29)	

Abbreviations: FOLFOXIRI-B, fluorouracil, folinic acid, oxaliplatin, and irinotecan plus bevacizumab; IQR, interquartile range; mCRC, metastatic colorectal cancer.

^a^ Data are presented as number (percentage) of patients unless otherwise indicated.

^b^ χ^2^ Tests were performed to compare oncologists who did and did not discuss FOLFOXIRI-B with patients except where otherwise indicated.

^c^ *t* Test.

^d^ Seven academic university hospitals and 1 specialized cancer center in the Netherlands.

^e^ Fisher exact test (performed between subgroups academic vs teaching or community hospital).

### Discussion of FOLFOXIRI-B With Patients

Fourteen medical oncologists (14%) reported that they did not discuss FOLFOXIRI-B with their patients. These medical oncologists were older (median [interquartile range] age, 53 [38-56] years) and more often male (12 of 14 [86%]) compared with medical oncologists who did discuss FOLFOXIRI-B as a treatment option (median [interquartile range] age, 42 [38-50] years; 37 of 87 male [43%]) (Table 3). Reported reasons not to discuss the therapy option with eligible patients are presented in Table 4. Eight of these 14 medical oncologists (57%) declared that the interview was a reason to include FOLFOXIRI-B as a treatment option for future patients. A total of 65 medical oncologists (75%) who discuss FOLFOXIRI-B with patients did not limit this treatment option to one specific subgroup of patients (ie, patients with potentially resectable metastases, requiring tumor reduction, having tumors with a *BRAF*^V600^ and/or *RAS *variant, or with sidedness) (Figure, A). Medical oncologists who were selective in their discussions limited the treatment option to the following: patients with an ECOG PS of 0 or 1 (55 medical oncologists [63%]), only patients requiring tumor reduction (ie, threatening tumor location, large tumor load, or high symptom burden) (30 [34%]), only patients who had tumors with a *BRAF*^V600^ and/or *RAS* variant (16 [19%]), and only patients with potentially resectable metastases (14 [16%]). A total of 47 medical oncologists (54%) reported comorbidity as a reason that they would allow oxaliplatin-containing doublets plus bevacizumab but not FOLFOXIRI-B. In all, 70 of 73 medical oncologists (96%) claimed that the COVID-19 pandemic had no effect on FOLFOXIRI-B prescription rates in the 4 months before the study week (eTable 2 in Supplement 1).

**Table 4.** Medical Oncologists’ Perspectives

Current clinical practice and science integration regarding FOLFOXIRI-B	No. (%) of medical oncologists
Medical oncologists who discuss FOLFOXIRI-B as a first-line treatment option with patients with mCRC (N = 101)	87 (86)
Events that motivated medical oncologists to discuss FOLFOXIRI-B as a treatment option with patients in clinical practice (n = 77)	
TRIBE (2014 and 2015), TRIBE2 (2020), and/or meta-analysis by Cremolini et al^4^	23 (30)
Conference or refresher course	21 (27)
Contact with a thought leader	16 (21)
Recommendation in Dutch guideline (2017) or NVMO committee	15 (20)
Other	2 (3)
Reported level of comfort in communicating FOLFOXIRI-B as a first-line treatment option to patients (n = 87)	
Comfortable	72 (83)
Somewhat comfortable	14 (16)
Challenging	1 (1)
Reasons not to feel totally comfortable (n = 15)^a^	
Doubts about effectiveness or whether effectiveness outweighs toxicity	14 (80)
Prefers to withhold oxaliplatin or irinotecan for second-line treatment	5 (33)
Frequency of required hospital visits	3 (20)
Being inexperienced with regard to this treatment option	2 (13)
Unknown effect on quality of life	1 (6)
Increased risk of induced neutropenia during COVID-19 pandemic	1 (6)
Other	2 (13)
Medical oncologists who prefer an oxaliplatin-based first-line treatment over an irinotecan-based treatment (N = 101)	83 (82)
Medical oncologists who have prescribed FOLFOXIRI-B (n = 87)	74 (85)
Medical oncologists who aim to prescribe (n = 86)	
8 Cycles of first-line FOLFOXIRI-B	50 (58)
12 Cycles of first-line FOLFOXIRI-B	36 (42)
Perception of FOLFOXIRI-B toxicity in clinical practice (n = 73)	
As expected	37 (51)
Less than expected	32 (44)
Worse than expected	4 (6)
Medical oncologists who are aware of the content of TRIBE (2014-2015),^2,3^ TRIBE2 (2020),^4^ or meta-analysis by Cremolini et al^9^ (N = 101)	55 (54)
Medical oncologists who are aware of the Dutch guideline recommendations regarding FOLFOXIRI-B (2017) (N = 101)	38 (38)
Medical oncologists who are familiar with the positive NVMO committee FOLFOXIRI-B recommendation (approximately December 2020) (n = 91)	51 (56)

Abbreviations: FOLFOXIRI-B, fluorouracil, folinic acid, oxaliplatin, and irinotecan plus bevacizumab; mCRC, metastatic colorectal cancer; NVMO, Dutch Society of Medical Oncology; TRIBE, Combination Chemotherapy and Bevacizumab as First-Line Therapy in Treating Patients With Metastatic Colorectal Cancer; TRIBE2 (Upfront FOLFOXIRI Plus Bevacizumab and Reintroduction After Progression Versus mFOLFOX6 Plus Bevacizumab Followed by FOLFIRI Plus Bevacizumab in the Treatment of Patients With Metastatic Colorectal Cancer).

^a^ Multiple answer options. Therefore, percentages do not total 100%.

**Figure.**
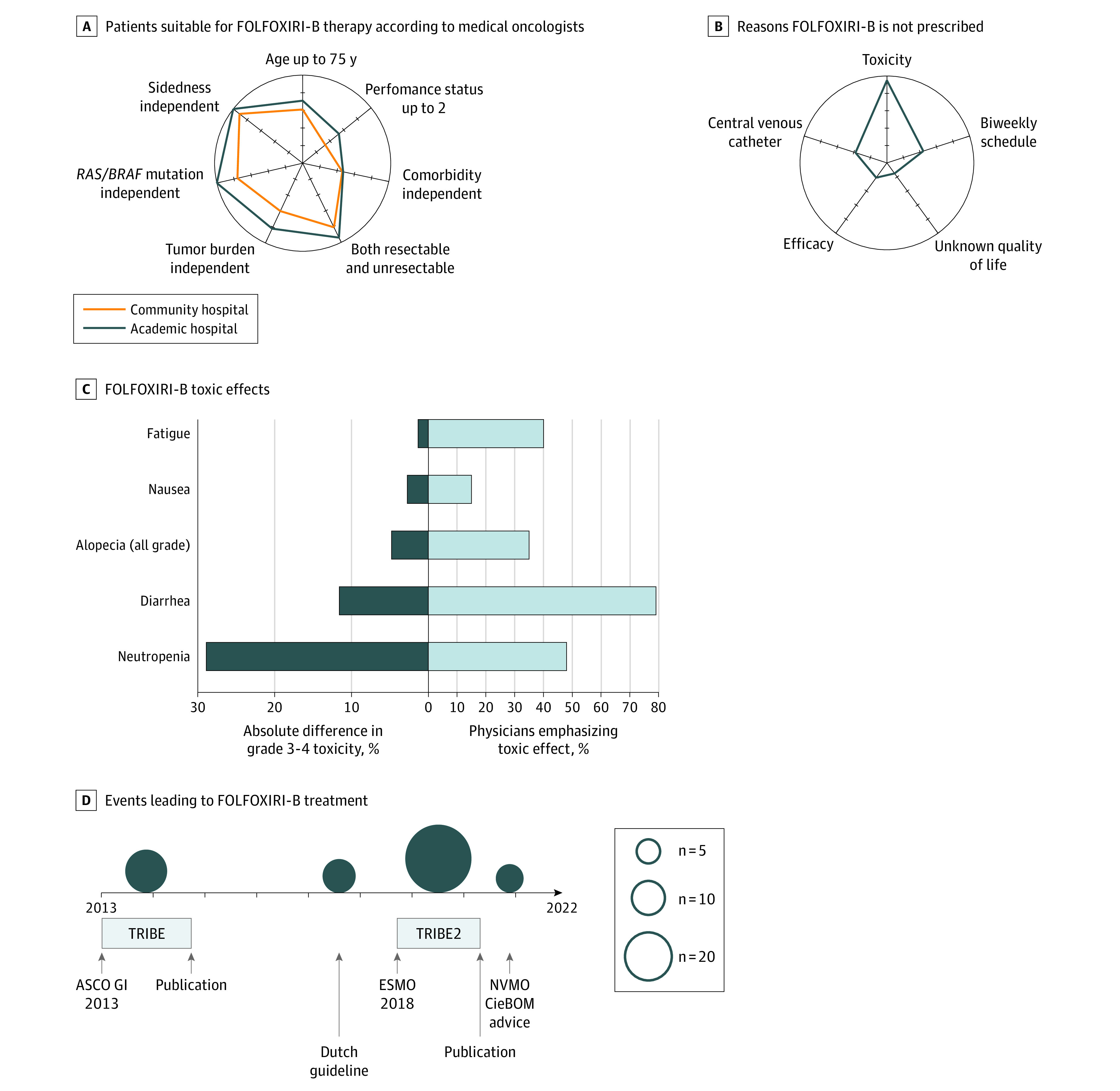
Interview Answers on Science Integration and Daily Practice A and B, Radar charts presenting answers to questions 11e and 11j of the interview script, respectively. Questions and answers are presented in eTable 2 and eFigure 2 in Supplement 1. In these radar charts, answer options are presented at each axis. All axes have the same origin, presenting the value of 0%. The end of every spoke presents 100%. The charts show the percentage of medical oncologists who claimed the answer presented on each axis. The farther toward the edge of a spoke, the higher the quantity of medical oncologists who claimed this answer. C, Fluorouracil, folinic acid, oxaliplatin, and irinotecan plus bevacizumab (FOLFOXIRI-B) toxic effects. On the left side of the graph, absolute differences in grades 3 to 4 adverse events (for alopecia, all grades) between oxaliplatin, fluorouracil, folinic acid, and bevacizumab (FOLFOX-B) and FOLFOXIRI-B are presented based on the results of the TRIBE2 (Upfront FOLFOXIRI Plus Bevacizumab and Reintroduction After Progression Versus mFOLFOX6 Plus Bevacizumab Followed by FOLFIRI Plus Bevacizumab in the Treatment of Patients With Metastatic Colorectal Cancer) trial. On the right side of the graph, answers to question 11k are shown (ie, percentages of medical oncologists who claim to emphasize the mentioned toxic effect when discussing FOLFOXIRI-B with patients). D, Answers to question 11a: events that led to implementation of FOLFOXIRI-B in daily practice. This figure represents a timeline. Spheres represent medical oncologists who claim to be influenced by the presented event(s) (text with arrows). The size of the spheres represents the absolute number of medical oncologists. ASCO GI 2013 indicates American Society of Clinical Oncology 2013 Gastrointestinal Cancers Symposium; ESMO 2018, European Society for Medical Oncology 2018 Congress; NVMO CieBOM, Nederlandse Vereniging voor Medische Oncologie Commissie ter Beoordeling van Oncologische Middelen; and TRIBE, Combination Chemotherapy and Bevacizumab as First-Line Therapy in Treating Patients With Metastatic Colorectal Cancer.

Reasons why patients refrain from treatment with FOLFOXIRI-B, according to medical oncologists, are presented in the Figure, B. Toxic effects were most reported. Adverse effects that are emphasized by medical oncologists are presented in the Figure, C. Of the 86 medical oncologists who answered the question, diarrhea (68 [79%]), neutropenia (41 [48%]), fatigue (34 [40%]), and alopecia (30 [35%]) were reported most frequently (eTable 2 in Supplement 1). Thirty-two of 73 medical oncologists (44%) with experience prescribing FOLFOXIRI-B in clinical care reported that treatment was better tolerated than first expected, whereas 4 (6%) reported worse tolerability than first anticipated.

Among 85 medical oncologists, 70 (82%) agreed with the statement, “I leave the therapy choice up to the patient. For patients the survival benefit of FOLFOXIRI-B does often not outweigh the disadvantages.” A total of 47 of 85 (55%) agreed with the statement, “I often indicate my preference for treatment with chemotherapy doublets with or without a biological instead of FOLFOXIRI-B. Patients follow that recommendation.” Finally, 60 of 86 (70%) agreed with the statement, “To a young, fit, and motivated patient, I indicate my preference for first-line treatment with FOLFOXIRI-B.”

### Integration of Scientific Knowledge

To assess scientific knowledge integration, we asked questions regarding awareness of FOLFOXIRI-B as a treatment option (Table 4 and eTable 2 in Supplement 1). A total of 55 of 101 medical oncologists (54%) were familiar with the contents of a research-related publication (TRIBE,^2,3^ TRIBE2,^4^ or meta-analysis^9^), 38 (38%) were familiar with the Dutch guideline recommendation on FOLFOXIRI-B (2017), and 51 (56%) were familiar with the positive recommendation of the NVMO committee that was published in December 2020.

Medical oncologists reported the following events that led to incorporation of FOLFOXIRI-B into their clinical practice (Figure, D): TRIBE,^2,3^ TRIBE2,^4^ or meta-analysis^9^ publication (23 [30%]), conference or refresher course attendance (21 [27%]), contact with an opinion leader (16 [21%]), and the 2017 Dutch guideline recommendation and/or 2020 NVMO committee recommendation (15 [20%]).

Medical oncologists who discuss FOLFOXIRI-B compared with medical oncologists who did not discuss this treatment option were more often aware of the outcome of trials regarding FOLFOXIRI-B (52 of 87 [60%] vs 3 of 14 [21%], *P* = .01) and the NVMO committee recommendation (47 of 78 [60%] vs 4 of 13 [31%], *P* = .047) but not the Dutch guideline recommendation. Medical oncologists who did not discussed FOLFOXIRI-B had significantly lower FAS scores (mean [SD], 4.2 [2.8] vs 2.6 [2.3]; *P* = .04). A low FAS score was correlated with agreement with the statement, “I often indicate my preference for treatment with chemotherapy doublets with or without a biological instead of FOLFOXIRI-B. Patients follow that recommendation” (mean [SD], 5.0 [3.1] vs 3.6 [2.6]; *P* = .03).

## Discussion

In this cross-sectional study, the current, first-line FOLFOXIRI-B prescription rate was 9% compared with 2% in 2015 to 2018. Although treatment with FOLFOXIRI-B has increased, our results indicate that only 1 in 7 estimated eligible patients is being treated with FOLFOXIRI-B. Considering that patients with a watchful waiting approach are not part of this study and that approximately 25% of patients with mCRC in the Netherlands never receive systemic therapy,^21^ our definition for estimated eligibility is in line with a previous estimate of 40%.^22^

Why were 6 of 7 eligible patients treated with oxaliplatin-based doublets instead of the more efficacious FOLFOXIRI-B? We evaluated medical oncologists’ perspectives and experiences. Our results indicate that a high percentage (86%) of medical oncologists discuss FOLFOXIRI-B with eligible patients. Moreover, most Dutch medical oncologists do not exclude specific patients outside what is defined by the TRIBE trials’ eligibility criteria. However, reasons for the discrepancy between eligible patients and treated patients with FOLFOXIRI-B might partly lie in patient-physician communication. In our sample, 14% of interviewed medical oncologists never discuss FOLFOXIRI-B in clinical practice. Although most medical oncologists reported discussing FOLFOXIRI-B with eligible patients, 56% also report that they regularly communicate a preference for a chemotherapy doublet to these patients. In addition, oncologists reported that they were more inclined to discuss FOLFOXIRI-B with patients with potentially resectable metastases, younger patients, and patients with more aggressive disease, such as patients with *BRAF^V600^*-variant tumors. The previously mentioned reasons might explain the observed difference between the proportion of patients with whom FOLFOXIRI-B was discussed and the patients who actually received this treatment.

The assumption that FOLFOXIRI-B might be more efficacious for patients with *BRAF^V600^*-variant tumors is based on the post hoc subgroup analysis of the updated TRIBE trial, which was subsequently incorporated in clinical guidelines.^2,3^ However, a recent subgroup analysis^9^ of individual patient data, which included 1697 patients, concluded that no increased benefit is observed for patients with *BRAF^V600^*-variant tumors. This meta-analysis^9^ also assessed whether treatment benefit was larger for patients with liver-only metastasis. In addition, although R0 resections were more frequent (16.4% vs 11.8%), no significant interaction was found between treatment with FOLFOXIRI-B and the achievement of R0 resection in terms of overall survival.

When FOLFOXIRI-B is discussed as a treatment option, there are reasonable arguments as to why a patient might choose to forgo treatment with FOLFOXIRI-B. Medical oncologists reported increased toxic effects as the major reason for patients to decline FOLFOXIRI-B treatment, followed by the more frequent hospital visits from the 2-weekly schedule and the necessity of a central venous catheter. The discussion between oncologists and patients on the possible benefits and burden of a specific treatment can be challenging. When the condition of the patient allows several treatment options, personal beliefs, cultural aspects, and expectations of both physician and patient play a role. The goal should be that information provided to patients is based on objective data to allow patients to make a choice that has the best chance of meeting personal goals.

Indeed, the adverse effects that are discussed by medical oncologists in our study are mostly in accordance with the TRIBE2 trial.^4^ However, it is unknown how strongly toxic effects are emphasized by medical oncologists in their discussion with patients. In this respect, 44% of medical oncologists report to have initially overestimated the toxic effects for patients compared with 6% of medical oncologists who reported an initial underestimation when prescribing FOLFOXIRI-B. This finding suggests that Dutch medical oncologists might overemphasize toxic effects in daily practice.

Another factor related to the relatively low adoption rate might be the transfer of scientific advancement to daily practice. Typically, knowledge is generated through clinical studies. Published results are subsequently included in the synthesized literature base and then broadly disseminated through conferences, workshops, and continuing medical education. Finally, when incorporated as standard of care, said insights may ultimately lead to improved health outcomes. Several estimates have suggested that this entire process takes up to 17 years to complete.^23,24,25,26^ Considering the current rate of the development of novel and effective cancer treatments, the adoption of these treatments in daily practice should be much improved. Our data indicate that medical oncologists gained knowledge on the benefit of FOLFOXIRI-B through a various number of sources, if at all. We found that medical oncologists who were aware of more literature on FOLFOXIRI-B seemed to be less inclined to communicate a preference for treatment with chemotherapy doublets to a patient, resulting in patients following that recommendation. This finding could imply that a higher level of awareness of medical oncologists leads to a more unbiased discussion of treatment options with patients.

Continuing medical education, feedback on guideline adherence, modular updates of guidelines, and more frequent dissemination of novel, positive developments through authorized organizations should aid in this process. These types of interventions are known to correspond with guideline dissemination and implementation with significant positive changes in patient outcomes.^27^ In addition, real-life data should provide up-to-date information on the implementation of novel, effective treatments in daily practice and will provide more transparency on the quality of cancer care in individual hospitals. Flash mob studies, such as the current study, may be useful in achieving this goal.

This study is, to our knowledge, the sixth and largest flash mob study ever performed worldwide.^13,14,15,16,17^ Ultrafast data generation coupled with thoroughly preplanned data analysis enabled us to provide a true snapshot of current medical practice.

### Limitations

Although this is the largest flash mob study to date, the results are based on a subset of medical institutions that were willing to participate. The proportion of nonparticipating academic, teaching, and nonteaching hospitals was balanced, suggesting that this bias might be limited. Furthermore, the patients currently treated with FOLFOXIRI-B and the number of medical oncologists who did not discuss FOLFOXIRI-B was small, reducing statistical power for group comparison. In this study, we estimated eligibility for FOLFOXIRI-B based on the TRIBE inclusion and exclusion criteria. The true proportion of eligible patients might be slightly different considering that we did not take into account specific contraindications to irinotecan and the complex ECOG PS criteria for the 70- to 75-year age group. This study was performed during the COVID-19 pandemic. Although 96% of participating medical oncologists reported no change in prescription habits during the study period, it is possible that the pandemic has influenced the adoption rate of FOLFOXIRI-B. With respect to the interviews taken, interviewers were trained, and the questions had prespecified answer options. Nonetheless, interviewer bias could have occurred. In addition, these results should be interpreted within the geographic context of the study. The Dutch FOLFOXIRI-B guideline recommendations are similar to international recommendations. However, variation in prescription rates in other countries based on cultural differences is conceivable (ie, emphasis on quality of life vs duration of life as the primary treatment goal).^28^

## Conclusions

This cross-sectional study found that the actual adoption rate of FOLFOXIRI-B as first-line treatment for patients with mCRC in the Netherlands remains relatively low despite the fact that most medical oncologists report discussing this treatment option with their patients. The results of this study provide insight into underlying reasons and offer guidance to accelerate the incorporation of knowledge into clinical practice for the benefit of patients.

## Notes

Supplement 1. **eMethods.** Definitions and Assumptions **eTable 1.** Baseline Characteristics of the 2 Populations **eTable 2.** Questions and Answers From Interviews With Medical Oncologists **eFigure 1. **Flow Diagram Data Sources **eFigure 2. **Flowchart Interview Script

Supplement 2. **Nonauthor Collaborators.** EXCITE Study Group Members

## References

[1] Van Cutsem E, Cervantes A, Adam R, . ESMO consensus guidelines for the management of patients with metastatic colorectal cancer. Ann Oncol. 2016;27(8):1386-1422. doi:10.1093/annonc/mdw235 27380959

[2] Loupakis F, Cremolini C, Masi G, . Initial therapy with FOLFOXIRI and bevacizumab for metastatic colorectal cancer. N Engl J Med. 2014;371(17):1609-1618. doi:10.1056/NEJMoa1403108 25337750

[3] Cremolini C, Loupakis F, Antoniotti C, . FOLFOXIRI plus bevacizumab versus FOLFIRI plus bevacizumab as first-line treatment of patients with metastatic colorectal cancer: updated overall survival and molecular subgroup analyses of the open-label, phase 3 TRIBE study. Lancet Oncol. 2015;16(13):1306-1315. doi:10.1016/S1470-2045(15)00122-9 26338525

[4] Cremolini C, Antoniotti C, Rossini D, . Upfront FOLFOXIRI Plus Bevacizumab and Reintroduction After Progression Versus mFOLFOX6 Plus Bevacizumab Followed by FOLFIRI Plus Bevacizumab in the Treatment of Patients With Metastatic Colorectal Cancer (TRIBE2): a multicentre, open-label, phase 3, randomised, controlled trial. Lancet Oncol. 2020;21(4):497-507. doi:10.1016/S1470-2045(19)30862-9 32164906

[5] Van Cutsem E, Cervantes A, Nordlinger B, Arnold D; ESMO Guidelines Working Group. Metastatic colorectal cancer: ESMO Clinical Practice Guidelines for diagnosis, treatment and follow-up. Ann Oncol. 2014;25(July)(suppl 3):iii1-iii9. doi:10.1093/annonc/mdu260 25190710

[6] Benson AB III, Venook AP, Cederquist L, . Colon cancer, version 1.2017: clinical practice guidelines in oncology. J Natl Compr Canc Netw. 2017;15(3):370-398. doi:10.6004/jnccn.2017.0036 28275037

[7] Tanis PJ, Beets GL, Verhoef C, et al. Dutch Colorectal Cancer Guideline. 2014. Accessed February 25, 2021. https://richtlijnendatabase.nl/richtlijn/colorectaal_carcinoom_crc/startpagina_-_crc.html

[8] NVMO. Expert opinion: update guideline colorectal cancer limited to topics that have a direct impact on general practice and based on expert opinion. 2017. Accessed February 25, 2021. https://www.nvmo.org/wp-content/uploads/2018/07/Update_richtlijn_CRC_sept_2017.pdf

[9] Cremolini C, Antoniotti C, Stein A, . Individual patient data meta-analysis of FOLFOXIRI plus bevacizumab versus doublets plus bevacizumab as initial therapy of unresectable metastatic colorectal cancer. J Clin Oncol. 2020;38(28):JCO2001225. doi:10.1200/JCO.20.01225 32816630

[10] NVMO-Commissie BOM. FOLFOXIRI plus bevacizumab als eerstelijnsbehandeling met herintroductie bij gemetastaseerd colorectaal carcinoom. December 2020:37-42. Accessed February 25, 2021. https://www.nvmo.org/bom/folfoxiri-plus-bevacizumab-als-eerstelijnsbehandeling-met-herintroductie-bij-gemetastaseerd-colorectaal-carcinoom

[11] Merriam-Webster. *Flash mob* definition. Accessed February 25, 2021. https://www.merriam-webster.com/dictionary/flash%20mob

[12] Stassen PM, Cals JWL. Flashmobstudies: wetenschap in een flits. Ned Tijdschr Geneeskd. 2020;164:1-5. Accessed August 24, 2021. https://www.ntvg.nl/artikelen/flashmobstudies-wetenschap-een-flits 32608930

[13] Semler MW, Stover DG, Copland AP, . Flash mob research: a single-day, multicenter, resident-directed study of respiratory rate. Chest. 2013;143(6):1740-1744. doi:10.1378/chest.12-1837 23197319 PMC3747725

[14] Alsma J, van Saase JLCM, Nanayakkara PWB, ; FAMOUS Study Group. the power of flash mob research: conducting a nationwide observational clinical study on capillary refill time in a single day. Chest. 2017;151(5):1106-1113. doi:10.1016/j.chest.2016.11.035 27940191

[15] Wesselius HM, van den Ende ES, Alsma J, ; “Onderzoeks Consortium Acute Geneeskunde” Acute Medicine Research Consortium. Quality and quantity of sleep and factors associated with sleep disturbance in hospitalized patients. JAMA Intern Med. 2018;178(9):1201-1208. doi:10.1001/jamainternmed.2018.2669 30014139 PMC6142965

[16] Schols AMR, Willemsen RTA, Bonten TN, . A nationwide flash-mob study for suspected acute coronary syndrome. Ann Fam Med. 2019;17(4):296-303. doi:10.1370/afm.2401 31285206 PMC6827655

[17] Nicolson P, Perry R, Fisher A. A HaemSTAR-led, UK-wide ‘flash-mob ’audit of intravenous immunoglobulin use in immune thrombocytopenia. Clin Med (Lond). 2019;19(suppl 3):82-83. doi:10.7861/clinmedicine.19-3-s8230651254

[18] von Elm E, Altman DG, Egger M, Pocock SJ, Gøtzsche PC, Vandenbroucke JP; STROBE Initiative. The Strengthening the Reporting of Observational Studies in Epidemiology (STROBE) statement: guidelines for reporting observational studies. Bull World Health Organ. 2007;85(11):867-872. doi:10.2471/BLT.07.045120 18038077 PMC2636253

[19] Mekenkamp LJM, Koopman M, Teerenstra S, . Clinicopathological features and outcome in advanced colorectal cancer patients with synchronous vs metachronous metastases. Br J Cancer. 2010;103(2):159-164. doi:10.1038/sj.bjc.6605737 20551951 PMC2906733

[20] Goey KKH, Sørbye H, Glimelius B, . Consensus statement on essential patient characteristics in systemic treatment trials for metastatic colorectal cancer: supported by the ARCAD Group. Eur J Cancer. 2018;100:35-45. doi:10.1016/j.ejca.2018.05.010 29936065

[21] Hamers PAH, Elferink MAG, Stellato RK, . Informing metastatic colorectal cancer patients by quantifying multiple scenarios for survival time based on real-life data. Int J Cancer. 2021;148(2):296-306. doi:10.1002/ijc.33200 32638384 PMC7754475

[22] Cremolini C. When to use triplet chemotherapy as first-line treatment in metastatic colorectal cancer. Clin Adv Hematol Oncol. 2019;17(8):433-435. 31449510

[23] Green LW, Ottoson JM, García C, Hiatt RA. Diffusion theory and knowledge dissemination, utilization, and integration in public health. Annu Rev Public Health. 2009;30:151-174. doi:10.1146/annurev.publhealth.031308.100049 19705558

[24] Morris ZS, Wooding S, Grant J. The answer is 17 years, what is the question: understanding time lags in translational research. J R Soc Med. 2011;104(12):510-520. doi:10.1258/jrsm.2011.110180 22179294 PMC3241518

[25] Balas EA, Boren SA. Managing clinical knowledge for health care improvement. Yearb Med Inform. 2000;09(1):65-70. doi:10.1055/s-0038-1637943 27699347

[26] Grant J, Cottrell R, Cluzeau F, Fawcett G. Evaluating “payback” on biomedical research from papers cited in clinical guidelines: applied bibliometric study. BMJ. 2000;320(7242):1107-1111. doi:10.1136/bmj.320.7242.1107 10775218 PMC27352

[27] Tomasone JR, Kauffeldt KD, Chaudhary R, Brouwers MC. Effectiveness of guideline dissemination and implementation strategies on health care professionals’ behaviour and patient outcomes in the cancer care context: a systematic review. Implement Sci. 2020;15(1):41. doi:10.1186/s13012-020-0971-6 32493348 PMC7268663

[28] Glynne-Jones R, Harrison M. FOLFOXIRI reintroduction in metastatic colorectal cancer. Lancet Oncol. 2020;21(4):468-469. doi:10.1016/S1470-2045(20)30087-5 32164907

