# Supplementary material for: Trends in Use and Perceptions About Triplet Chemotherapy Plus Bevacizumab for Metastatic Colorectal Cancer
Source: JAMA Netw Open. 2021 Sep 10;4(9):e2124766. doi: 10.1001/jamanetworkopen.2021.24766 (PMC8433607; doi:10.1001/jamanetworkopen.2021.24766)
Supplement: Supplement 1. — eMethods. Definitions and Assumptions eTable 1. Baseline Characteristics of the 2 Populations eTable 2. Questions and Answers From Interviews With Medical Oncologists eFigure 1. Flow Diagram Data Sources eFigure 2. Flowchart Interview Script [file jamanetwopen-e2124766-s001.pdf]

Supplementary Online Content

van Nassau SC, Bond MJ, Scheerman I, et al; EXCITE (From Clinical Trial to Bedside: Triplet Chemotherapy in Metastatic Colorectal Cancer) Study Group. Trends in use and perceptions about triplet chemotherapy plus bevacizumab for metastatic colorectal cancer. *JAMA Netw Open*. 2021;4(9):e2124766. doi:10.1001/jamanetworkopen.2021.24766

eMethods. Definitions and Assumptions

eTable 1. Baseline Characteristics of the 2 Populations

eTable 2. Questions and Answers From Interviews With Medical Oncologists

eFigure 1. Flow Diagram Data Sources

eFigure 2. Flowchart Interview Script

This supplementary material has been provided by the authors to give readers additional information about their work.

© 2021 van Nassau SC et al. *JAMA Network Open.*

eMethods. Definitions and assumptions

**Definitions and assumptions regarding first line systemic treatment for metastatic colorectal cancer (stage IV, M1)**
Systemic treatment includes chemotherapy and/or targeted therapy (cetuximab or panitumumab, and bevacizumab).
HIPEC (intraperitoneal hyperthermic chemoperfusion) and chemoradiation were not considered first line systemic chemotherapy.
Both systemic treatment before or after metastasectomy and palliative systemic therapy were regarded as systemic therapy given in the context of metastases.
In our retrospective cohort from the Dutch National Cancer Registry, first line systemic treatment was defined as:<br>- All systemic agents that were:<br>o started before the first systemic agent was stopped AND<br>o started within 56 days of the start date of the first agent (i.e. before first evaluation) AND<br>o not chemoradiation
In our 2020-2021 cohort, first line systemic treatment was defined as:<br>- All agents that were administered as a systemic chemotherapy for metastatic colorectal cancer (M1) at the first treatment cycle. Capecitabine monotherapy as part of chemoradiation was not considered first line treatment
We pooled patients that were treated with FOLFOXIRI with and without bevacizumab. As a consequence, when we speak of FOLFOXIRI-B in the main text, we mean both FOLFOXIRI with and without bevacizumab.
**Definitions and assumptions regarding baseline characteristics**
Adjuvant chemotherapy was defined as exposure to oxaliplatin prior to the diagnosis of metastatic colorectal cancer. If adjuvant systemic therapy was administered less than 6 months before occurrence of metastatic disease (n=3), it was not counted as a prior treatment regimen for metastatic disease.
Synchronous metastasis was defined as metastases diagnosed within 6 months following colorectal cancer (CRC) diagnosis (date of pathology results that confirmed colon and rectal adenocarcinoma)[1]
Metachronous metastasis was defined as metastases diagnosed more than 6 months after the CRC diagnosis (date of pathology results that confirmed colon and rectal adenocarcinoma)[1]
The diagnostic date of metastatic colorectal cancer was considered the date of confirming pathology results or, in case no tissue was collected, the date on which metastases were visible on imaging for the first time
Primary tumor location was categorized as right-sided colon (caecum up to and including transverse colon), left-sided colon (splenic flexure up to and including sigmoid) or rectum (rectosigmoid to rectum).
BRAF mutation was defined as the presence of V600E/K. If a different BRAF mutation was present the patient was considered to be BRAF wildtype. If a patient had a RAS mutation and BRAF was not tested, BRAF was also considered to be wildtype.[2]
RAS mutation was defined as the presence of a pathologic KRAS, HRAS or NRAS mutation
**Definitions and assumptions regarding treatment groups**
Intensive chemotherapy (table 1) was defined as CAPOX/FOLFOX/FOLFIRI with or without a biological or irinotecan monotherapy
Monotherapy (table 1) was defined as S1/capecitabine/5FU with or without a biological, and immune checkpoint inhibition
Estimated eligibility for FOLFOXIRI-B was defined as; patients ≤75 years of age, with an ECOG performance status of 0-2, who were treated with FOLFOXIRI or oxaliplatin-doublets (i.e., FOLFOX or CAPOX) with or without bevacizumab outside of a clinical trial
**Definitions and assumptions regarding scientific knowledge integration**
We created the FOLFOXIRI-B Awareness Scale (FAS); a score that represents awareness of medical oncologists on knowledge regarding FOLFOXIRI-B. 1 point was attributed for:<br>- Knowledge on the content of TRIBE 1 (2014 and 2015)[3,4]<br>- Knowledge on the content of TRIBE 2 (2020)[5]<br>- Knowledge on the content of the meta-analysis by Cremolini et al. (2020)[6]<br>- Knowledge on mentioning of FOLFOXIRI-B in Dutch clinical guidelines.[7,8] If, in addition, an oncologist was aware of the Dutch guideline recommendation (2017), 2 more points were attributed<br>- Knowledge regarding the publication of a NVMO committee recommendation (December 2020).[9] If a medical oncologist claimed to be aware of the publication, but stated a false publication date (before 2020), 1 point was deducted. However, if a medical oncologist was truly aware of the positive recommendation, 2 more points were attributed.<br>The minimum score was -1 and the maximum score 9

eReferences

1. Goey KKH, Sørbye H, Glimelius B, et al. Consensus statement on essential patient characteristics in systemic treatment trials for metastatic colorectal cancer: Supported by the ARCAD Group. *Eur J Cancer*. 2018;100:35-45. doi:10.1016/j.ejca.2018.05.010
2. Smeby J, Sveen A, Merok MA, et al. CMS-dependent prognostic impact of KRAS and BRAFV600E mutations in primary colorectal cancer. *Ann Oncol*. 2018;29(5):1227-1234. doi:10.1093/annonc/mdy085
3. Loupakis F, Cremolini C, Masi G, et al. TRIBE: Initial Therapy with FOLFOXIRI and Bevacizumab for Metastatic Colorectal Cancer. *N Engl J Med*. 2014;371(17):1609-1618. doi:10.1056/nejmoa1403108
4. Cremolini C, Loupakis F, Antoniotti C, et al. TRIBE update: FOLFOXIRI plus bevacizumab versus FOLFIRI plus bevacizumab as first-line treatment of patients with metastatic colorectal cancer: Updated overall survival and molecular subgroup analyses of the open-label, phase 3 TRIBE study. *Lancet Oncol*. 2015;16(13):1306-1315. doi:10.1016/S1470-2045(15)00122-9
5. Cremolini C, Antoniotti C, Rossini D, et al. TRIBE2: Upfront FOLFOXIRI plus bevacizumab and reintroduction after progression versus mFOLFOX6 plus bevacizumab followed by FOLFIRI plus bevacizumab in the treatment of patients with metastatic colorectal cancer (TRIBE2): a multicentre, open-label, phase. *Lancet Oncol*. 2020;21(4):497-507. doi:10.1016/S1470-2045(19)30862-9
6. Cremolini C, Antoniotti C, Stein A, et al. Meta-analyse TRIBE: Individual patient data meta-analysis of folfoxiri plus bevacizumab versus doublets plus bevacizumab as initial therapy of unresectable metastatic colorectal cancer. *J Clin Oncol*. 2020;38(28):3314-3324. doi:10.1200/JCO.20.01225
7. Dutch colorectal cancer guideline. Dutch colorectal cancer guideline. Published online 2014.
8. Expert Opinion NVMO. Update guideline colorectal cancer limited to topics that have a direct impact on general practice and based on expert opinion. Published online 2017. doi:DOI:
9. NVMO-commissie BOM. FOLFOXIRI plus bevacizumab als eerstelijnsbehandeling met herintroductie bij gemetastaseerd colorectaal carcinoom. 2020;(december):37-42.

© 2021 van Nassau SC et al. *JAMA Network Open.*

eTable 1. Baseline Characteristics of the 2 Populations

**Patient characteristics**	**2020-2021 (n=282)**	**2015-2018 (n=5948)**
Age, median years [IQR]	66 [57-73]	66 [57-73]
≤ 75	222 (79%)	4995 (84%)
> 75	60 (21%)	953 (16%)
Sex		
Male	164 (58%)	3503 (59%)
Female	118 (42%)	2445 (41%)
WHO Performance Status		
0/1	235 (83%)	3537 (59%)
2	26 (9.2%)	397 (6.7%)
>2	8 (2.8%)	57 (1.0%)
Missing	13 (4.6%)	1957 (33%)
Tumour site		
Right sided	98 (35%)	2081 (35%)
Left sided or rectum	177 (63%)	3712 (62%)
Unknown/overlapping	7 (2.5%)	155 (2.6%)
Resected primary tumour		
Yes	113 (40%)	2073 (35%)
No	169 (60%)	3875 (65%)
Time to metastases		
Synchronous	207 (73%)	5948 (100%)
Metachronous	75 (27%)	0[a]
Liver only		
Yes	70 (25%)	2277 (38%)
No	212 (75%)	3671 (62%)
BRAF status		
Wild type	168 (60%)	2168 (36%)
Mutated	26 (9.2%)	392 (6.6%)
Missing	88 (31%)	3388 (57%)
RAS status		
Wild type	91 (32%)	1434 (24%)
Mutated	104 (37%)	1514 (26%)
Missing	87 (31%)	3000 (50%)
Microsatellite status		
MSS / proficient MMR	194 (69%)	2466 (42%)
MSI / deficient MMR	11 (3.9%)	124 (2.1%)
Missing	77 (27%)	3358 (56%)
Prior adjuvant chemotherapy (CAPOX/FOLFOX)	23 (8.2%)	0[a]
Systemic first-line therapy		
CAP/5-FU mono +/- B	54 (19%)	1281 (22%)
CAPOX/FOLFOX +/-B	184 (65%)	4286 (72%)
FOLFIRI +/- B	11 (3.9%)	49 (0.8%)

© 2021 van Nassau SC et al. *JAMA Network Open.*

	2020-2021 (n=282)	2015-2018 (n=5948)
FOLFOXIRI +/- B	25 (8.9%)	142 (2.4%)
Other	8 (2.8%)	190 (3.2%)
First-line systemic therapy was administered within clinical trial	27 (9.6%)	439 (7.4%)

Data are n (%) unless otherwise specified.
IQR, interquartile range; MMR, mismatch repair; MSI, microsatellite instable; MSS, microsatellite stable; WHO, World health organization.
[a]The retrospective NCR (Netherlands Cancer Registry) cohort exists only of synchronous metastatic colorectal cancer patients

© 2021 van Nassau SC et al. *JAMA Network Open.*

eTable 2. Questions and Answers From Interviews With Medical Oncologists

**Integration of scientific knowledge**	**Number of medical oncologists**	
	**Yes (%)**	**No or don't know (%)**
1 to 4. Questions regarding baseline characteristics: Age, gender, work setting, experience years and number of treated mCRC patients/year		
5. Has the COVID pandemic affected your ability to keep up-to-date with new developments in oncology care? (n=101)	34 (34%)	67 (66%)
Positively (more time)	9 (26%)	
Negatively (less time)	25 (74%)	
6. Are you aware of the content of publications comparing FOLFOXIRI-B with FOLFIRI-B/FOLFOX-B – open question (n=101)	55 (55%)	46 (45%)
Medical oncologists that are aware of the content of the TRIBE 1 trial (n=54)	37 (69%)	17 (31%)
Medical oncologists that report the TRIBE 1 trial changed their practice most	13 (35%)	24 (65%)
Medical oncologists that are aware of the content of the TRIBE 2 trial (n=54)	43 (80%)	11 (20%)
Medical oncologists that report the TRIBE 2 trial changed their practice most	21 (49%)	22 (51%)
Medical oncologists that are aware of the content of the meta-analysis by Cremolini et al (n=54)	20 (37%)	34 (63%)
Medical oncologists that report the meta-analysis changed their practice most	6 (30%)	14 (70%)
7. Do you know whether FOLFOXIRI-B is mentioned in the current Dutch guideline? (n=101)	54 (54%)	47 (46%)
7a. If yes, is FOLFOXIRI-B recommended as a first-line treatment option for metastatic colorectal cancer patients? (n=54)	38 (70%)	16 (30%)
7b. Do you prescribe FOLFOXIRI-B according to the current Dutch clinical guidelines? (n=38)	19 (50%)	19 (50%)
8. Do you know if the NVMO committee has published a recommendation regarding FOLFOXIRI-B (n=91)	62 (68%)	29 (32%)
8a. Medical oncologists that correctly stated the positive NVMO committee recommendation (approx. December 2020) (n=62)	51 (82%)	11 (18%)
8b. If familiar, did this NVMO committee recommendation change your practice? (n=50)	17 (34%)	33 (66%)
**Clinical practice**	**Mentioned by n (%) medical oncologists**	
9. How is 5-FU administered to patients in your hospital? (n=101)		
With a FOLFusor pump through a central venous line at home	97 (96%)	
Intravenously during a 48h hospital admission	4 (4%)	
10. Do you have a preference for an oxaliplatin or irinotecan based first-line systemic therapy? (n=101)		
Oxaliplatin	83 (82%)	
Irinotecan	0	
No preference	18 (18%)	
11. Do you discuss FOLFOXIRI-B as a first-line treatment with mCRC patients? (n=101)		
Yes --> continue to 11a.	87 (86%)	
No --> continue to 13. (see S2)	14 (14%)	

© 2021 van Nassau SC et al. *JAMA Network Open.*

Clinical practice	Mentioned by n (%) medical oncologists
11a. After which event did you decide to start discussing FOLFOXIRI-B as a treatment option with patients in clinical care? (n=77)	
Publication trial (TRIBE 1 2014 and update2015, TRIBE 2 2020, meta-analysis Cremolini 2020)	23 (30%)
After visiting a conference/attending a refresher course	21 (27%)
After speaking with a thought leader	9 (12%)
Due to treatment recommendation in Dutch guideline (2017)	8 (10%)
Due to NVMO committee recommendation (2020)	7 (9.1%)
During oncology fellowship	7 (9.1%)
Trial participation in which FOLFOXIRI-B is a treatment arm (2018)	2 (2.6%)
11b. Do you feel comfortable when discussing FOLFOXIRI-B as a first-line treatment option with mCRC patients? (n=87)	
Comfortable --> continue to 11e.	72 (83%)
Somewhat comfortable --> continue to 11c.	14 (16%)
Not comfortable --> continue to 11c.	1 (1.1%)
11c. What causes you to feel not/moderately comfortable?[a] (n=15)	
Doubts whether effectiveness outweighs toxicity	12 (80%)
Prefers to withhold oxaliplatin or irinotecan for second line treatment	5 (33%)
Frequency of required hospital visits	3 (20%)
Doubts about effectiveness	2 (13%)
Being unexperienced regarding this therapy	2 (13%)
Unknown effect on quality of life	1 (6.5%)
Increased risk of induced neutropenia during COVID pandemic	1 (6.5%)
Fear that patients will underestimate toxicity and overestimate effectiveness	1 (6.5%)
External validity of the TRIBE studies for the Dutch population is limited	1 (6.5%)
11d. How many months of median overall survival gain would make you feel more comfortable? (n=7)	
>4 months	4 (57%)
≤4 months	3 (43%)
11e. Which patients do you discuss FOLFOXIRI-B with?[a] (n=87)	
Age plays a role, I use the age limit below	63 (72%)
- <75 years	34 (54%)
- <70 years	23 (37%)
- <65 years	6 (9.5%)
Performance status plays a role, I discuss the treatment option with	87 (100%)
- Patients with WHO performance status 0-1	55 (63%)
- Patients with WHO performance status 0-2	18 (21%)
- WHO PS 0 when 70-75 years old and WHO PS ≤2 if <70 years	14 (16%)
There is comorbidity for which I do discuss oxaliplatin-doublets but no FOLFOXIRI (with or without bevacizumab)	47 (54%)
Resectability plays an important role: I only discuss FOLFOXIRI-B with patients with potentially resectable colorectal metastases	14 (16%)
The necessity of tumor reduction plays an important role: I only discuss FOLFOXIRI-B with patients with a threatening tumor location, large tumor load or high symptom burden	30 (35%)
Molecular classification (BRAF, RAS) plays an important role: I only discuss FOLFOXIRI-B with patients with a specific mutation	16 (19%)
- BRAF mutation	14 (88%)
- BRAF ánd RAS mutation	2 (12%)

© 2021 van Nassau SC et al. *JAMA Network Open.*

Clinical practice	Mentioned by n (%) medical oncologists	
Sidedness plays an important role: I only discuss FOLFOXIRI-B with patients with right sided tumors	6 (7.0%)	
11f. Have you prescribed FOLFOXIRI-B to mCRC patients? (n=87)		
Yes	74 (85%)	
No	13 (15%)	
11g. For oncologists in teaching and community hospitals: did the recommendation of an academic center ever play a role in the choice to prescribe FOLFOXIRI-B? (n=60)		
Yes	22 (37%)	
No	38 (63%)	
11h. Would/do you aim for 8 or 12 cycles first-line FOLFOXIRI-B if toxicity permits? (n=86)		
8 cycles	50 (58%)	
12 cycles	36 (42%)	
**11i. Statements on discussing FOLFOXIRI-B and subsequent treatment choice**	**Agree**	**Disagree**
"I leave the therapy choice up to the patient. For patients the survival benefit of FOLFOXIRI-B does often not outweigh the disadvantages" (n=85)	70 (82%)	15 (18%)
"I often indicate my preference for treatment with chemotherapy doublets with or without a biological instead of FOLFOXIRI-B. Patients follow that recommendation." (n=85)	47 (55%)	37 (44%)
"To a young, fit and motivated patient, I indicate my preference for first-line treatment with FOLFOXIRI-B" (n=86)	60 (70%)	26 (30%)
**Clinical practice**	**Mentioned by n (%) medical oncologists**	
11j. When you discuss FOLFOXIRI-B with a patient, what are reasons to choose a different intensive chemotherapy regimen?[a] (open question, n=86)		
Toxicity/effect on overall condition	82 (95%)	
2 weekly schedule	38 (44%)	
Necessity of central line	33 (38%)	
Limited survival gain	18 (21%)	
Unknown impact on quality of life	13 (15%)	
Maintenance with 5-FU-B instead of CAP-B	5 (5.8%)	
Prefers to withhold oxaliplatin or irinotecan for second-line treatment	3 (3.5%)	
Other/not classified	2 (2.3%)	
COVID pandemic	0	
11k. What side effects do you emphasize when discussing FOLFOXIRI-B in comparison with CAPOX or FOLFOX with bevacizumab?[a] (open question, n=86)		
Diarrhea	68 (79%)	
Neutropenia	41 (48%)	
Fatigue	34 (40%)	
Alopecia	30 (35%)	
Nausea and vomiting	13 (15%)	
Overall discomfort	9 (10%)	
Necessity of a central line	8 (9.3%)	
Neuropathy	5 (5.8%)	

© 2021 van Nassau SC et al. *JAMA Network Open.*

Clinical practice	Mentioned by n (%) medical oncologists
Other/not classified	4 (4.7%)
I emphasize no extra side effects	2 (2.3%)
11l. What is your perception of FOLFOXIRI-B toxicity in clinical practice? (n=73)	
Tolerability as expected	37 (51%)
Tolerability better than expected	32 (44%)
Tolerability worse than expected	4 (5.5%)
12. Do you believe the COVID pandemic has had any influence on your FOLFOXIRI-B prescription rates specifically in the last 4 months? (n=73)	
Yes, it has been a reason to refrain from this treatment option	3 (4.1%)
No, it did not have any influence	70 (96%)
13. Did you gain knowledge about FOLFOXIRI-B due to 'events' other than the TRIBE publications, the Dutch guideline or NVMO committee recommendation? (n=14)	
No	4 (29%)
Yes, at a conference	5 (36%)
Yes, by conversation with a colleague	2 (14%)
Yes, other	3 (21%)
14. What is the reason to refrain from discussing FOLFOXIRI-B as a first-line treatment option with patients?[a] (open question, n=13)	
Toxicity outweighs effectiveness	10 (77%)
Prefers to withhold oxaliplatin or irinotecan for second line treatment	5 (38%)
Not convinced by effectiveness	4 (31%)
My patients are not suitable candidates	3 (10%)
The frequency of necessary hospital visits	2 (15%)
Quality of life data is still missing	1 (8.0%)
Unaware of this treatment option	0
Due to the COVID pandemic	0
15. How many months of median overall survival gain would be sufficient to start discussing FOLFOXIRI-B as a first-line treatment option? (open question, n=12)	
>4 months	8 (67%)
≤4 months	4 (33%)
16. What side effects are a reason to prefer doublet chemotherapy with bevacizumab over treatment with FOLFOXIRI-B?[a] (open question, n=14)	
Diarrhea	6 (43%)
No side effects, but the overall impact (central line, 2 weekly schedule)	4 (29%)
All side-effects but more intense	4 (29%)
Neutropenia	4 (29%)
Fatigue	1 (7.1%)
Alopecia	2 (14%)
Nausea and vomiting	0
Neuropathy	0
17. Do you intent to discuss the treatment option henceforth? (n=13)	
Yes	8 (62%)
No	5 (38%)

COVID, corona virus disease; mCRC, metastatic colorectal cancer; NVMO committee, Dutch society of medical oncology; WHO, world health organization; PS, performance status

[a] Multiple answer options. Therefore percentages do not add up to 100%

© 2021 van Nassau SC et al. *JAMA Network Open.*

eFigure 1. Flow Diagram Data Sources

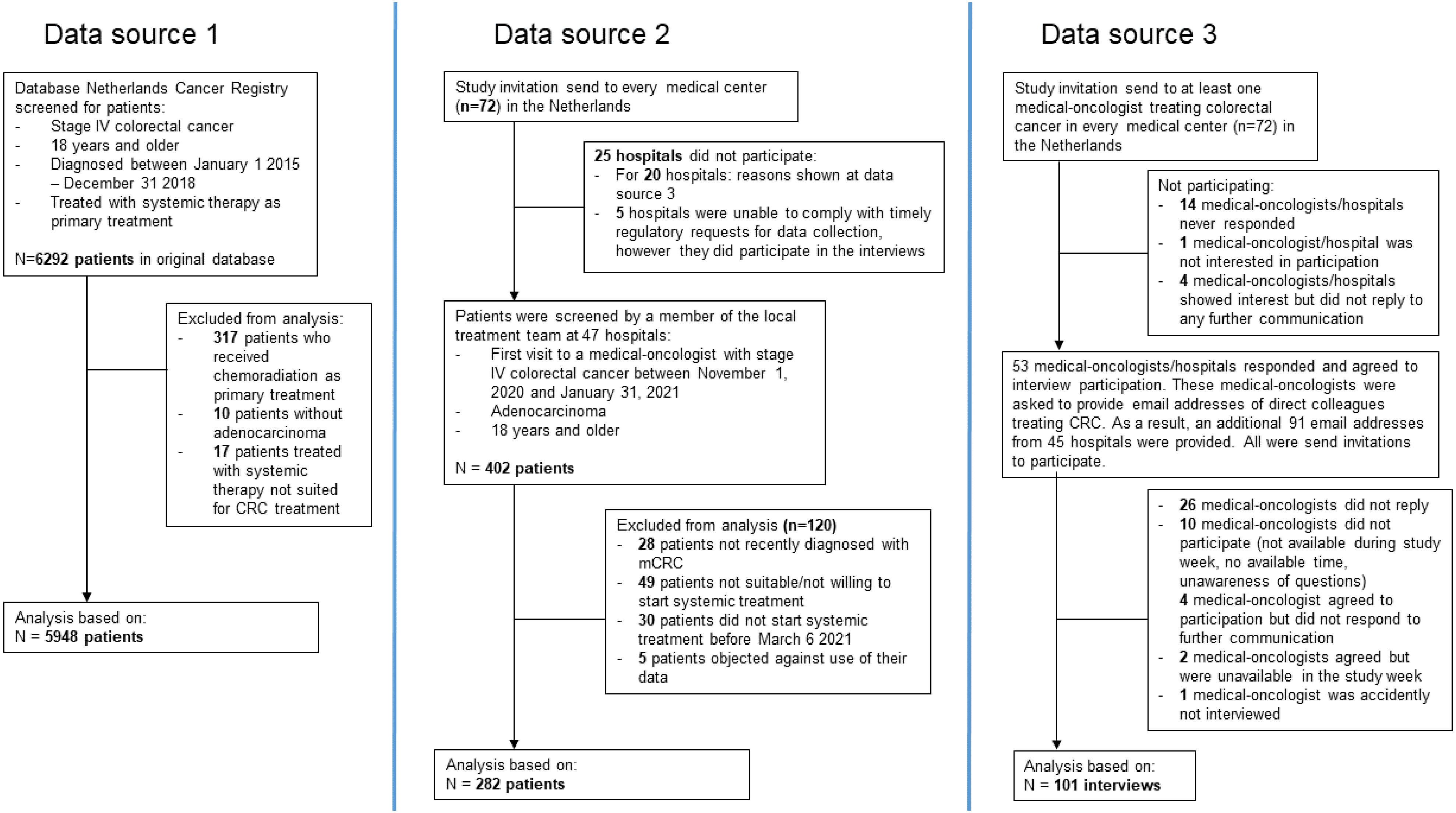

© 2021 van Nassau SC et al. *JAMA Network Open.*

eFigure 2. Flowchart Interview Script

All questions and answers of the interview script are presented in eTable 2

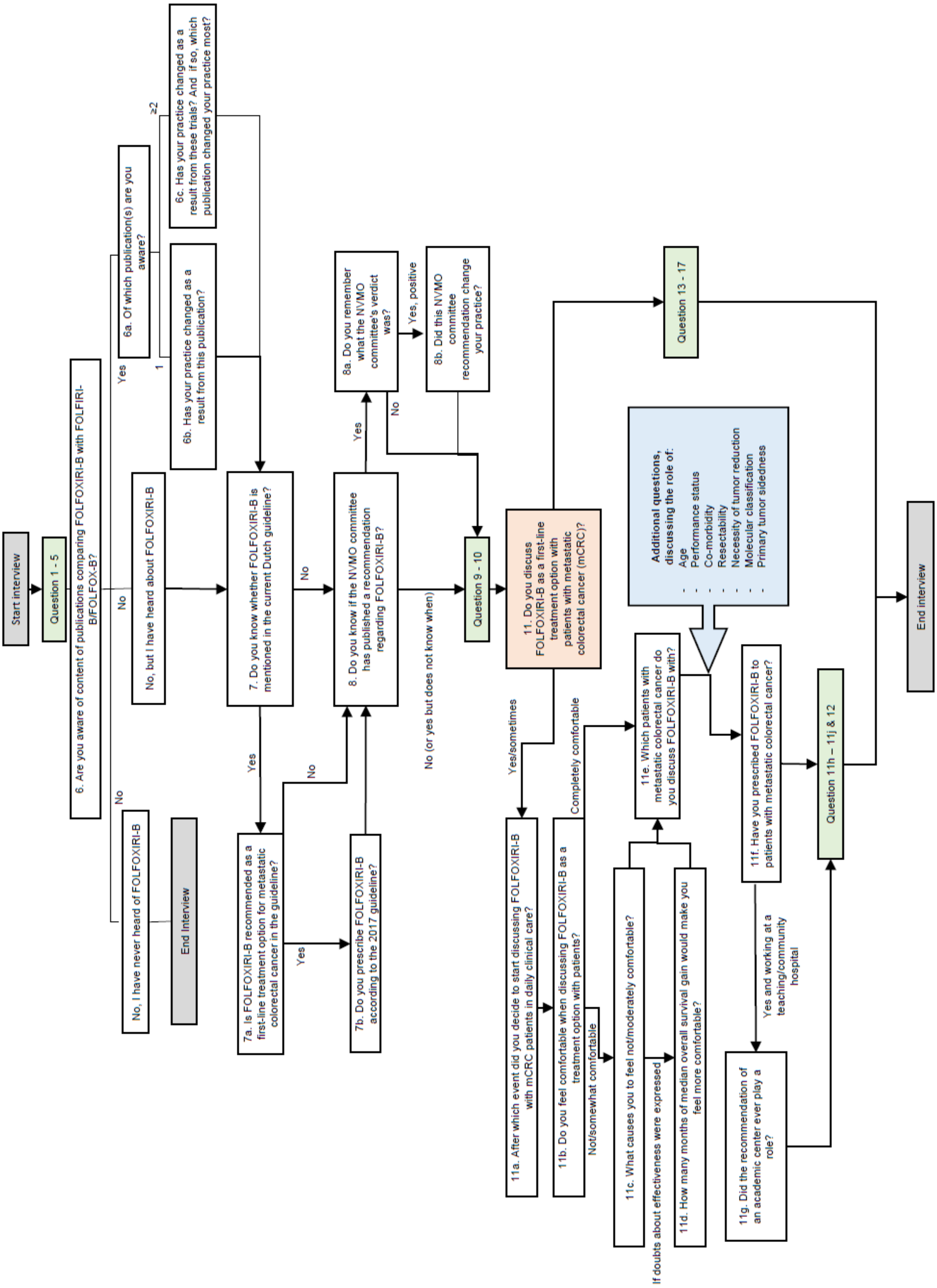

© 2021 van Nassau SC et al. *JAMA Network Open.*
