# Supplementary material for: Trends in Use and Perceptions About Triplet Chemotherapy Plus Bevacizumab for Metastatic Colorectal Cancer
Source: JAMA Netw Open. 2021 Sep 10;4(9):e2124766. doi: 10.1001/jamanetworkopen.2021.24766 (PMC8433607; doi:10.1001/jamanetworkopen.2021.24766)
Supplement: Supplement 2. — Nonauthor Collaborators. EXCITE Study Group Members [file jamanetwopen-e2124766-s002.pdf]

Supplemental Online Content: Nonauthor Collaborators

*Indicates required information. Only first name, last name, and suffix will appear in PubMed.

*Group Name(s): EXCITE Study Group							
***First Name and Middle Initial(s)**	***Last Name**	***Suffix (eg, Jr, III)**	Academic Degrees	Institution	Location (city, state/province, country)	Role or Contribution, eg, chair, principal investigator	Group (if more than 1 Group listed in the byline) and/or Subgroup (eg, Steering Committee)
Mirte M	Streppel		MD, PhD	Department of Medical Oncology, Amsterdam University Medical Centre	Amsterdam, The Netherlands		
Leon	van Hout		BSc	Department of Medical Oncology, Amsterdam University Medical Centre	Amsterdam, The Netherlands		
Maartje	Los		MD, PhD	Department of Medical Oncology, St Antonius Hospital	Nieuwegein, The Netherlands		
Zonne	Hofman		MD, PhD	Department of Medical Oncology, St Antonius Hospital	Nieuwegein, The Netherlands		
Lonneke W	Kessels		MD	Department of Medical Oncology, Deventer Hospital	Deventer, The Netherlands		
Erica HA	Groen			Department of Medical Oncology, Deventer Hospital	Deventer, The Netherlands		
Lieke LH	van Huis-Tanja		MD, PhD	Department of Medical Oncology, Diakonessenhuis	Utrecht, The Netherlands		
Felix E	de Jongh		MD, PhD	Department of Medical Oncology, Ikazia Hospital	Rotterdam, The Netherlands		
Louise JM	Alferink		MD, PhD	Department of Medical Oncology, Ikazia Hospital	Rotterdam, The Netherlands		
Hans-Martin MB	Otten		MD, PhD	Department of Medical Oncology, Meander Medical Centre	Amersfoort, The Netherlands		
Els JE	Wink-van Gestel			Department of Medical Oncology, Meander Medical Centre	Amersfoort, The Netherlands		
Ankie MT	van der Velden		PhD	Department of Medical Oncology, Tergooi Hospital	Hilversum, The Netherlands		
Dirkje W	Sommeijer		MD, PhD	Department of Medical Oncology, Flevo Hospital	Almere, The Netherlands		

Supplemental Online Content: Nonauthor Collaborators

*Indicates required information. Only first name, last name, and suffix will appear in PubMed.

*First Name and Middle Initial(s)	*Last Name	*Suffix (eg, Jr, III)	Academic Degrees	Institution	Location (city, state/province, country)	Role or Contribution, eg, chair, principal investigator	Group (if more than 1 Group listed in the byline) and/or Subgroup (eg, Steering Committee)
Sadaf Oliai	Araghi		MSc, PhD	Department of Medical Oncology, Flevo Hospital	Almere, The Netherlands		
Loes M	Latten-Jansen		MD	Dept. of Internal Medicine, Division of Medical Oncology, GROW - School for Oncology and Developmental Biology, Maastricht University Medical Center	Maastricht, the Netherlands		
Milou S	Keusters		BSc	Department of Medical Oncology, Netherlands Cancer Institute	Amsterdam, The Netherlands		
Brigitte C M	Haberkorn		MD	Department of Medical Oncology, Maasstad Hospital	Rotterdam, The Netherlands		
Arie J	Verschoor		MD	Department of Medical Oncology, Reinier de Graaf gasthuis	Delft, The Netherlands		
Carolien	Haazer		MSc	Department of Medical Oncology, Reinier de Graaf Gasthuis	Delft, The Netherlands		
Geert-Jan	Creemers		MD, PhD	Department of Medical Oncology, Catharina Hospital	Eindhoven, The Netherlands		
Nikki FT	Henckens		BSc	Department of Medical Oncology, Catharina Hospital	Eindhoven, The Netherlands		
Frank JF	Jeurissen		MD, PhD	Department of Medical Oncology, Medical Centre Haaglanden	Den Haag, The Netherlands		
Karin EM	de Nijs		BSc	Department of Medical Oncology, Medical Centre Haaglanden	Den Haag, The Netherlands		
Ronald	Hoekstra		MD	Department of Medical Oncology, Hospital Group Twente	Hengelo, The Netherlands		
Judith J	Zwartjens		MD	Department of Medical Oncology, Hospital Group Twente	Almelo, The Netherlands		
Mathijs P	Hendriks		MD	Department of Medical Oncology, Northwest Clinics	Alkmaar, The Netherlands		

Supplemental Online Content: Nonauthor Collaborators

*Indicates required information. Only first name, last name, and suffix will appear in PubMed.

***First Name and Middle Initial(s)**	***Last Name**	***Suffix (eg, Jr, III)**	Academic Degrees	Institution	Location (city, state/province, country)	Role or Contribution, eg, chair, principal investigator	Group (if more than 1 Group listed in the byline) and/or Subgroup (eg, Steering Committee)
Arthur D	van Leeuwen		BSc	Department of Medical Oncology, Northwest Clinics	Alkmaar, The Netherlands		
Hester	van Cruijssen		MD, PhD	Department of Internal Medicine, Antonius Hospital	Sneek, The Netherlands		
Philo T	Werner		MD	Department of Medical Oncology, VieCuri Medical Centre	Venlo, The Netherlands		
Wendy ECJ	Heuts			Department of Medical Oncology, VieCuri Medical Centre	Venlo, The Netherlands		
Peter	Nieboer		MD	Department of Medical Oncology, Wilhelmina Hospital	Assen, The Netherlands		
Natascha AJB	Peters		MD	Department of Medical Oncology, St Jans Gasthuis	Weert, The Netherlands		
Marly	van Cranenbroek			Department of Medical Oncology, St Jans Gasthuis	Weert, The Netherlands		
Theo	van Voorthuizen		MD	Department of Medical Oncology, Rijnstate Hospital	Arnhem, The Netherlands		
Frederiek	Terheggen		MD	Department of Medical Oncology, Bravis Hospital	Roosendaal, The Netherlands		
Merel	Pieters		BSc	Department of Medical Oncology, Radboud University Medical Centre	Nijmegen, The Netherlands		
Mark PS	Sie		MD	Department of Medical Oncology, ZorgSaam Hospital	Terneuzen, The Netherlands		
Lieke HJ	Simkens		MD, PhD	Department of Medical Oncology, Maxima Medical Centre	Veldhoven, The Netherlands		
Julia GL	Olislagers		BSc	Department of Medical Oncology, Maxima Medical Centre	Veldhoven, The Netherlands		
Miriam L	Wumkes		MD, PhD	Department of Medical Oncology, Jeroen Bosch Hospital	's Hertogenbosch, The Netherlands		
Roel	Janssen		MD	Department of Medical Oncology, Jeroen Bosch Hospital	's Hertogenbosch, The Netherlands		

Supplemental Online Content: Nonauthor Collaborators

*Indicates required information. Only first name, last name, and suffix will appear in PubMed.

***First Name and Middle Initial(s)**	***Last Name**	***Suffix (eg, Jr, III)**	Academic Degrees	Institution	Location (city, state/province, country)	Role or Contribution, eg, chair, principal investigator	Group (if more than 1 Group listed in the byline) and/or Subgroup (eg, Steering Committee)
Leontine	Spierings		MD	Department of Medical Oncology, Alrijne hospital	Alphen aan den Rijn, The Netherlands		
Esther	van Staveren			Department of Medical Oncology, Alrijne Hospital	Alphen aan den Rijn, The Netherlands		
Iris	Kats		MD	Department of Medical Oncology, Medical Spectrum Twente	Enschede, The Netherlands		
Allert H	Vos		MD	Department of Medical Oncology, Bernhoven Hospital	Uden, The Netherlands		
Josca J	Heier		MSc	Department of Medical Oncology, Bernhoven Hospital	Uden, The Netherlands		
Edwin A	van Breugel		MD	Department of Internal Medicine, Dijklander Hospital	Hoorn, The Netherlands		
Jeroen	Vincent		MD	Department of Medical Oncology, Elkerliek Hospital	Helmond, The Netherlands		
Marjan A	Davidis		MD	Department of Medical Oncology, Beatrix Hospital,	Gorinchem, The Netherlands		
Teunie	Sepers			Department of Medical Oncology, Beatrix Hospital	Gorinchem, The Netherlands		
Johan JB	Janssen		MD	Department of Medical Oncology, Canisius-Wilhelmina Hospital	Nijmegen, The Netherlands		
Berend BO	Broeren		BSc	Department of Internal Medicine, Canisius-Wilhelmina Hospital	Nijmegen, The Netherlands		
Linda MH	van de Winkel		MD	Department of Medical Oncology, St Anna Hospital	Geldrop, The Netherlands		
Sieneke A	Hiddink		MD	Department of Internal Medicine, Gelre Hospital	Apeldoorn, The Netherlands		
Annemarie SB	Conijn-Mensink		MD	Department of Medical Oncology, Zaans Medical Centre	Zaandam, The Netherlands		
Stefanie	van Lunteren		MSc	Department of Medical Oncology, Zaans Medical Centre	Zaandam, The Netherlands		

Supplemental Online Content: Nonauthor Collaborators

*Indicates required information. Only first name, last name, and suffix will appear in PubMed.

***First Name and Middle Initial(s)**	***Last Name**	***Suffix (eg, Jr, III)**	Academic Degrees	Institution	Location (city, state/province, country)	Role or Contribution, eg, chair, principal investigator	Group (if more than 1 Group listed in the byline) and/or Subgroup (eg, Steering Committee)
Annette A	van Zweeden		MD	Department of Internal Medicine, Amstelland Hospital	Amstelveen, The Netherlands		
Magda	Vergouwe		BSc	Department of Medical Oncology, Amstelland Hospital	Amstelveen, The Netherlands		
Brian MJ	Scholtes		MD	Department of internal medicine, Pantein Hospital	Beugen, The Netherlands		
Serge E	Dohmen		MD, PhD	Department of Medical Oncology, BovenIJ Hospital	Amsterdam, The Netherlands		
Marcia	Ijzer			Department of Medical Oncology, BovenIJ Hospital	Amsterdam, The Netherlands		
Jaap	de Boer		MD	Department of Medical Oncology, Tjongerschans Hospital	Heerenveen, The Netherlands		
Kees CJA	Punt		MD, PhD	Department of Epidemiology, Julius Center for Health Sciences and Primary Care, University Medical Center	Utrecht, The Netherlands		
Geraldine R	Vink		MSc, PhD	Department of Medical Oncology, Imaging & Oncology, University Medical Center Utrecht	Utrecht, The Netherlands		
Patricia AH	Hamers		MD	Department of Medical Oncology, Imaging & Oncology, University Medical Center Utrecht	Utrecht, The Netherlands		
Karel C	Smit		MD	Department of Epidemiology, Julius Center for Health Sciences and Primary Care, University Medical Center	Utrecht, The Netherlands		
Maarten A	Huismans		MD	Department of Medical Oncology, Imaging & Oncology, University Medical Center Utrecht	Utrecht, The Netherlands		

Supplemental Online Content: Nonauthor Collaborators

*Indicates required information. Only first name, last name, and suffix will appear in PubMed.

***First Name and Middle Initial(s)**	***Last Name**	***Suffix (eg, Jr, III)**	Academic Degrees	Institution	Location (city, state/province, country)	Role or Contribution, eg, chair, principal investigator	Group (if more than 1 Group listed in the byline) and/or Subgroup (eg, Steering Committee)
Emerens GE	Wensink		MD	Department of Medical Oncology, Imaging & Oncology, University Medical Center Utrecht	Utrecht, The Netherlands		
Suzanna J	Schraa		MD	Department of Medical Oncology, Imaging & Oncology, University Medical Center Utrecht	Utrecht, The Netherlands		
Karlijn L	van Rooijen		MD	Department of Medical Oncology, Imaging & Oncology, University Medical Center Utrecht	Utrecht, The Netherlands		
Jeroen WG	Derksen		MSc, PhD	Julius Center for Health Sciences and Primary Care, University Medical Center Utrecht, Utrecht University	Utrecht, The Netherlands		
Anne M	May		MSc, PhD	Department of Epidemiology, Julius Center for Health Sciences and Primary Care, University Medical Center Utrecht, Utrecht University	Utrecht, The Netherlands		
Koen	Zwart		MD	Department of Medical Oncology, Imaging & Oncology, University Medical Center Utrecht	Utrecht, The Netherlands		
Jeanine MJ	Roodhart		MD, PhD	Department of Medical Oncology, Imaging & Oncology, University Medical Center Utrecht	Utrecht, The Netherlands		
